# Physicochemical properties and microbial community structure of the rhizosphere soil of *Cymbidium tracyanum*

**DOI:** 10.3389/fmicb.2025.1519614

**Published:** 2025-04-10

**Authors:** Wenwen Xie, Ying Tang, Haiming Li, Mingyu Dang, Jianuo Ci, Min Zheng, Erhao Zhang, Zhongbin Wang

**Affiliations:** ^1^School of Resources and Environment, Tibet Agriculture and Animal Husbandry College, Linzhi, China; ^2^College of Food Science, Tibet Agriculture and Animal Husbandry College, Linzhi, China

**Keywords:** *Cymbidium tracyanum* (*C. Tracyanum*), rhizosphere soil microorganisms, high-throughput sequencing, core microbes, soil physicochemical factors

## Abstract

To investigate the compositional characteristics of microbial communities in the rhizosphere soil of *Cymbidium tracyanum* (*C. tracyanum*) across different production regions in Tibet, as well as the correlation between these microorganisms and soil physicochemical factors, we analyzed soil microbial community composition in Bayi District, Chayu County, and Mêdog County in Linzhi City, Tibet, using Illumina MiSeq high-throughput sequencing technology. The results indicate that 7,467 bacterial operational taxonomic units (OTUs) and 2,866 fungal OTUs were identified in the rhizosphere soil of *C. tracyanum*. Significant differences were observed in the structural composition of bacterial and fungal communities across the different regions. The dominant bacterial phyla in the rhizosphere soil included Proteobacteria, Acidobacteria, Planctomycetota, and Firmicutes, while Ascomycota and Basidiomycota were the predominant fungal phyla. Additionally, variations in the physicochemical properties of the rhizosphere soil were observed among the different regions. Core microbiota analysis identified 214 core bacterial genera and 79 core fungal genera in the rhizosphere soil of *C. tracyanum* in Tibet. Correlation analysis revealed that changes in the core microbial community were associated with soil physicochemical factors to varying degrees, with total phosphorus and available phosphorus emerging as key factors influencing microbial diversity in the rhizosphere soil. In summary, the composition and diversity of bacterial and fungal communities in the rhizosphere soil of *C. tracyanum* varied across different production regions, and shifts in microbial community structure were closely linked to soil physicochemical factors.

## 1 Introduction

*Cymbidium tracyanum* (*C. tracyanum*) is a perennial epiphytic herb belonging to the genus *Cymbidium* within the Orchidaceae family. In China, it is primarily distributed in the southwestern part of Guizhou Province, the southwestern to southeastern regions of Yunnan Province, and the southeastern part of Tibet (China Flora Editorial Board, 1999). The leaves of *C. tracyanum* are long and evergreen, while its flowers are bold and magnificent, with a delicate fragrance. Due to its high ornamental and economic value, it is widely cultivated as a potted plant or cut flower (Chen et al., 2015; Li, 2014; Li et al., 2005; Lan et al., 2010; Wang et al., 2009). In recent years, excessive private harvesting has led to habitat destruction and a drastic decline in the population of *C. tracyanum*. Orchidaceae plants have unique biological characteristics, including small seeds lacking endosperm. Under natural conditions, their germination and seedling development depend on symbiotic fungi for nutrient acquisition. These fungi also colonize the root systems of adult plants. Orchidaceae plants propagate through either natural tillering or aseptic seeding. The former has a low reproductive coefficient, while the latter requires precisely determining the necessary nutritional conditions. Although *C. tracyanum* seedlings can now be obtained through aseptic seeding, the process remains labor-intensive, time-consuming, and costly. Furthermore, the quality of cultivated orchids is still inferior to that of their wild counterparts, with high transplant mortality rates, a high incidence of root rot, and difficulties in flowering and fruiting. These challenges limit the development of artificial cultivation and further exacerbate the overharvesting of wild orchids. Orchid plants are highly sensitive to environmental changes, and the gradual warming of the global climate poses additional threats to their growth. *C. tracyanum* is listed as a second-class protected plant in the *List of Nationally Protected Wild Plants*. However, research on *C. tracyanum* remains scarce both domestically and internationally. Existing studies primarily focus on its response mechanisms to environmental stress (Kuang and Zhang, 2015; Li and Zhang, 2016; Li et al., 2019) and tissue culture techniques (Chen et al., 2015; Li et al., 2005; Wang et al., 2009).

Soil is a crucial ecological component for plants, providing essential nutrients and water for growth. The microenvironment surrounding plant roots, known as the rhizosphere, was first described by German scientist Hiltner (Pahm et al., 2013; Zhang, 2020). Soil exhibits exceptionally high physicochemical heterogeneity at small scales (Vedere et al., 2022), contributing to complex and diverse biological communities (Burton et al., 2022). The formation of microbial communities in the rhizosphere is influenced by multiple factors, including soil composition, plant species, and plant genotype (Philippot et al., 2013; Trivedi et al., 2020). Since plants are immobile, the primary source of rhizosphere microorganisms is the indigenous microbial community present in the soil. The composition of this microbial reservoir is shaped by local climatical conditions, soil physicochemical properties, and biogeographical processes (Fierer and Jackson, 2006). In addition to soil microbes, microorganisms present in seeds also contribute to the rhizosphere microbial community assembly (Nelson, 2004). Consequently, there is growing interest in studying soil microbial diversity to leverage their ecological functions through research and sustainable management of this natural resource (Chandarana and Amaresan, 2022). Studies indicate that soil microbes are the most active biological component of soil, playing essential roles in nutrient cycling, energy flow, and various biochemical processes (Cartwright, 2014). Their secondary metabolites mediate communication, competition, and interactions among soil organisms and the surrounding environment (Crits-Christoph et al., 2018). The interaction between rhizosphere microbes and plants significantly influences material cycling and energy dynamics. Changes in microbial community composition and abundance can impact plant growth and development, flowering and fruiting, and plant interactions with phytophagous insects, all critical for plant health and yield (Sa et al., 2011; Wagg et al., 2011). Therefore, investigating the composition of rhizosphere microbial communities and their correlation with soil physicochemical factors is vital for genetically cultivating *C. tracyanum* and identifying beneficial microbial communities. Soil microorganisms are crucial in maintaining underground ecosystem functions (Evy et al., 2019). For instance, *Bacillus* species promote plant growth by producing bioactive compounds and enhance plant health by inhibiting pathogenic microbes (Fira et al., 2018). *Pseudomonas* is a typical rhizobacterium known for its plant growth-promoting properties (Berendsen et al., 2012). Wilkinson et al. isolated endophytic bacteria from 13 orchid species in Western Australia, with *Pseudomonas* being the most frequently identified genus (Wilkinson et al., 1994). Additionally, numerous studies have demonstrated that arbuscular mycorrhizal fungi form symbiotic associations with plant roots, contributing to sustainable ecosystem productivity by participating in host plant physiological and biochemical processes, regulating gene expression, enhancing nutrient absorption, and promoting grain formation and aboveground biomass accumulation (Zhang et al., 2013; Jy and Liu, 2024).

Changes in rhizosphere soil microbial communities are closely related to soil physicochemical factors. Studies have shown that soil microorganisms play an indispensable role in the biochemical cycling of elements such as carbon, nitrogen, and phosphorus and are crucial for maintaining the stability and health of soil ecosystems (Zhang et al., 2020). Research indicates that soil microorganisms can detect changes in soil properties caused by nitrogen fertilizers and respond differently (Zhao et al., 2014). Total nitrogen (TN) and organic matter significantly affect bacterial communities, and excessive nitrogen fertilizer use can lead to soil salinity accumulation, altering the bacterial composition in plant rhizosphere soil (Tayyab et al., 2019; Paterson et al., 2007).

Due to its low germination rate and high economic value, *C. tracyanum* has been overharvested, resulting in severe habitat destruction and a sharp decline in its population. Currently, no information is available on the characteristics of the *C. tracyanum* rhizosphere microbial community structure. This study examined the rhizosphere soil of *C. tracyanum* in three different regions of Tibet. High-throughput sequencing technology was used to analyze the microbial community composition and investigate its relationship with soil physicochemical factors. This study aims to provide a theoretical foundation for guiding the selection of beneficial microbial communities and improving the artificial cultivation of *C. tracyanum*.

## 2 Materials and methods

### 2.1 Study area overview

The study area is located in Linzhi City, Tibet Autonomous Region, and includes three sample sites: Bayi District (BY), Chayu County (CY), and Mêdog County (MT). The sampling site in BY is the Xizang Agricultural and Animal Husbandry University, which has a plateau temperate semi-humid monsoon climate with an annual average temperature of 8.5°C and an average annual precipitation of 654 mm. This site represents an artificial ecosystem. CY has a subtropical climate, with an annual average temperature of 12°C and an average annual precipitation of 801.1 mm, featuring a natural ecosystem. MT has a subtropical humid climate, with an average annual temperature of 18.4°C and an annual precipitation of 2,358 mm, representing a natural ecosystem. This study designated these three sampling sites as BY, CY, and MT. The latitude, longitude, and average altitude of each site are presented in Table 1.

**Table 1:** Basic survey of sample foundation.

Plot	BY	CY	MT
Longitude (E)	92°20′20″	97°46′ 67″	95°17′ 57″
Latitude (N)	29°40′06″	28°66′18 ″	29 °21′ 87″
Altitude (m)	2,941	2,800	1,200

### 2.2 Sample collection

In September 2023, following the principle of random multi-point mixing, healthy *C. tracyanum* orchids with consistent growth were selected from each test plot. Orchids that had not been exposed to chemical fertilizers or pesticides were collected. The root-shaking method collected rhizosphere soil samples, with three samples taken from each plot, amounting to 500 g. The collected soil was stored in sterile bags at a low temperature, transported to the laboratory, and processed within 24 h.

### 2.3 Measurement of rhizosphere soil physicochemical properties

The physicochemical properties of the soil were measured using conventional methods, following the procedures outlined by Bao (2000). The primary indicators analyzed included total phosphorus (TP), total potassium (TK), pH, TN, soil organic matter (SOM), available nitrogen (AN), available phosphorus (AP), and available potassium (AK). TP and AP were determined using the vanadium molybdate blue colorimetric method, while TK and AK were measured using a flame photometer. The pH value was determined using the electrode method. TN and AN were analyzed using the Kjeldahl method. SOM was determined using the potassium dichromate oxidation-external heating (volume) method.

### 2.4 Extraction of total DNA from rhizosphere soil samples and polymerase chain reaction (PCR) amplification

Total DNA was extracted from the soil samples using a Soil Genomic DNA Extraction Kit (DP336, Tiangen Biotech, Beijing, Co., Ltd., China). The quality of the extracted DNA was assessed using 0.8% agarose gel electrophoresis, while its concentration and purity were measured using a NanoDrop ND-2000 Ultraviolet-Vis Spectrophotometer (Thermo Scientific, Wilmington, USA). The extracted DNA was further examined using 1% agarose gel electrophoresis to confirm its mass and integrity. PCR amplification was performed to target variable regions using the primers 27F (5′-AGAGTTTGATCCTGGCTCAG-3′) and 1492R (5′-TACGG CTACCTTGTTACGACTT-3′). Additionally, the amplification of fungal internal transcribed spacer (ITS) sequences was conducted using the primers ITS1F (5′-CTTGGTCATTTAGAGGAAGTAA-3′) and ITS2R (5′-GCTGCGTTCTTCATCGATGC-3′). The amplification process began with an initial denaturation at 95°C for 3 min, followed by 35 cycles of denaturation at 95°C for 30 s, annealing at 55°C for 30 s, and extension at 72°C for 1 min and 30 s. A final extension was performed at 72°C for 10 min, and the samples were subsequently stored at 4°C. The PCR products were analyzed using 1% agarose gel electrophoresis. The amplification conditions followed the methodology described by Jy and Liu (2024). PCR products that met the quality requirements were excised from the gel, purified, and sent to Shanghai Majorbio Bio-Pharm Technology Co., Ltd. for high-throughput sequencing.

### 2.5 Statistical analysis of experimental data

Data organization and statistical analysis were conducted using Microsoft Excel 2010, while variance analysis was performed using Statistical Package for Social Sciences (version 26). One-way analysis of variance and Duncan’s multiple range test were used to determine significant differences in the data. The diversity and composition of soil microorganisms were analyzed using the i-Sanger platform^1^ provided by Shanghai Majorbio Bio-Pharm Technology Co., Ltd.

## 3 Results and analysis

### 3.1 Analysis of rhizosphere soil physicochemical properties at different sample sites

As shown in Table 2, the pH of rhizosphere soil across the three sample sites ranged from 4.69 to 5.93, indicating acidic conditions. There was no significant difference in pH between BY and CY. The TP and TK contents were significantly higher in MT samples than in CY and BY samples, while CY samples had the lowest TP and TK contents. The TN content was highest in CY samples, significantly exceeding that in MT and BY samples, with BY samples having the lowest TN content. The SOM content in CY samples was significantly higher than in MT and BY samples, whereas there was no significant difference between MT and BY. The AN content did not differ significantly between MT and CY samples but was significantly lower in BY samples. The AP content was highest in MT samples, followed by BY, and lowest in CY. The AK content was significantly higher in CY samples than in MT and BY samples, with no significant difference between MT and BY. These findings highlight variations in the physicochemical properties of rhizosphere soil among the three sample sites.

**Table 2:** Characteristics of physicochemical factors in the rhizosphere soil of *C. tracyanum*.

Parameters	Sample Sites		
	MT	CY	BY
TP (g/kg)	2.43 ± 0.076 a	0.56 ± 0.016 c	0.92 ± 0.025 b
TK (g/kg)	6.95 ± 0.264 a	3.39 ± 0.101 c	5.40 ± 0.370 b
pH	4.69 ± 0.111 b	5.66 ± 0.103 a	5.93 ± 0.046 a
TN (g/kg)	4.30 ± 0.089 b	6.00 ± 0.277 a	3.40 ± 0.185 c
SOM (g/kg)	96.93 ± 2.405 b	307.39 ± 6.529 a	107.20 ± 0.845 b
AN (g/kg)	0.56 ± 0.008 a	0.62 ± 0.040 a	0.33 ± 0.027 b
AP (mg/kg)	246.82 ± 10.274 a	30.88 ± 1.529 c	84.79 ± 1.048 b
AK (mg/kg)	111.69 ± 3.686 b	514.00 ± 16.371a	105.81 ± 4.595 b

Different lowercase letters indicate significant differences between samples at *p* < 0.05.

### 3.2 Sequencing results and diversity analysis of rhizosphere soil microbial samples

As shown in Table 3, 154,325 valid bacterial sequences were obtained from the rhizosphere soil across the three sample sites, with an average sequence length of 1,446 bp and sequencing coverage ranging from 97.22 to 97.83%. For fungi, 143,146 valid sequences were obtained, with an average sequence length of 607 bp and sequencing coverage ranging from 98.8 to 99.06%. These results indicate that the sequencing data accurately represent the microbial community composition and diversity at the three sample sites.

**Table 3:** Operational taxonomic units and related sequence indices in the rhizosphere soil of *C. tracyanum*.

Sample sites	Effective sequence number		Average length		Sequencing coverage		Number of OTUs	
	Bacteria	Fungi	Bacteria	Fungi	Bacteria	Fungi	Bacteria	Fungi
BY	56,926	45,166	1,449	601	97.54	98.93	2,766	1,108
CY	48,998	49,003	1,442	599	97.83	98.8	2004	1,077
MT	48,398	48,977	1,447	623	97.22	99.06	2,697	681

At a similarity threshold of 97.00%, 7,467 bacterial operational taxonomic units (OTUs) were identified, including 2,766 in BY samples, 2,004 in CY samples, and 2,697 in MT samples. Two thousand eight hundred sixty-six fungal OTUs were detected in the rhizosphere soil, with 1,108 in BY, 1,077 in CY, and 681 in MT samples. CY samples had the lowest number of OTUs, while BY samples had the highest.

**Figure 1:**
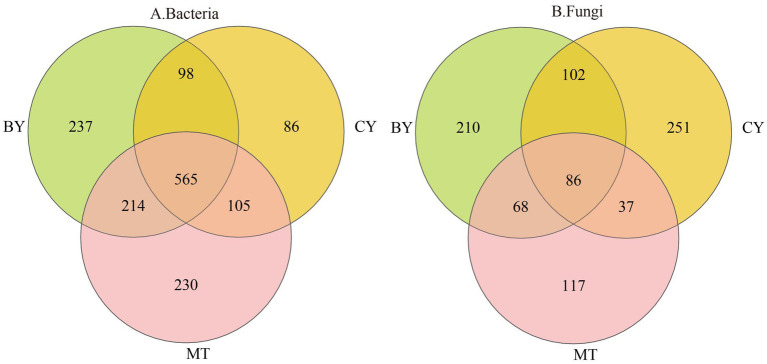
Venn diagrams of Bacteria **(A)**, Fungi **(B)** operational taxonomic units detected in the rhizosphere soil of *C. tracyanum*.

As illustrated in Figure 1A, 565 bacterial OTUs and 86 fungal OTUs were common across BY, CY, and MT samples, accounting for 7.56 and 3.00% of the total, respectively. The number of unique bacterial (Figure 1B) OTUs in BY, CY, and MT samples was 237, 86, and 230, respectively, while the number of unique fungal OTUs was 210, 251, and 117, respectively. These findings indicate variations in microbial community composition in the rhizosphere soil of *C. tracyanum* from different regions.

**Figure 2:**
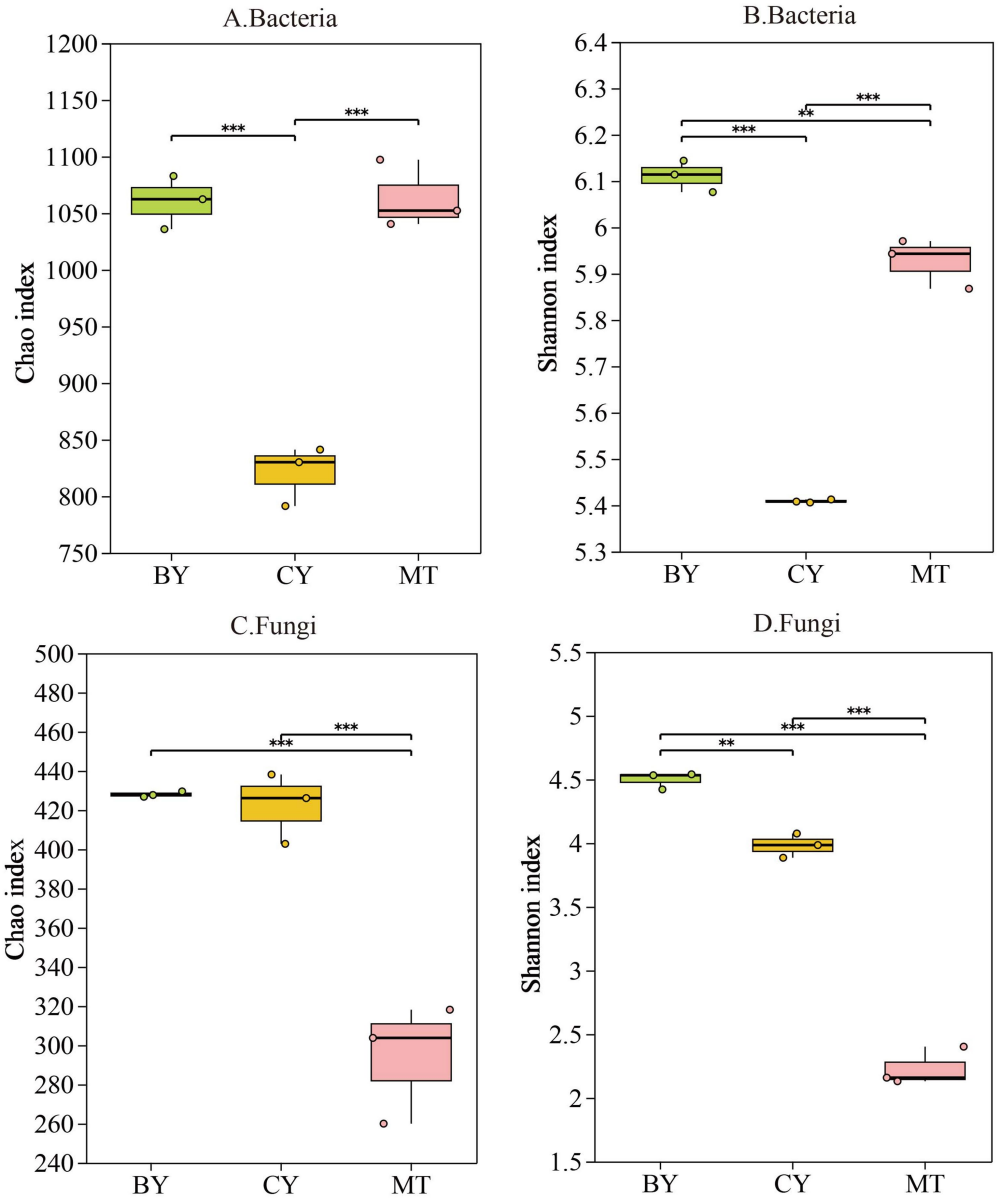
Alpha diversity indices of the microbial community in rhizosphere soil of *C. tracyanum*. Chao **(A,C)**. Shannon **(B,D)**.

Figure 2A,B shows that CY had the lowest bacterial abundance index among the three sample sites, while MT had the lowest fungal abundance index. In terms of bacterial diversity (Figure 2B,D), BY had the highest bacterial diversity index, followed by MT and CY. For fungal diversity, BY also exhibited the highest index, followed by CY, whereas MT had the lowest. CY had the lowest bacterial abundance and diversity indices, whereas MT had the lowest fungal abundance and diversity indices. The rhizosphere soil of BY exhibited the highest bacterial and fungal diversity indices, with fungal diversity significantly exceeding bacterial diversity. Similarly, BY samples had the highest bacterial and fungal abundance indices, with fungal abundance significantly surpassing bacterial abundance. These results indicate distinct microbial community compositions in the rhizosphere soil of *C. tracyanum* from different regions.

**Figure 3:**
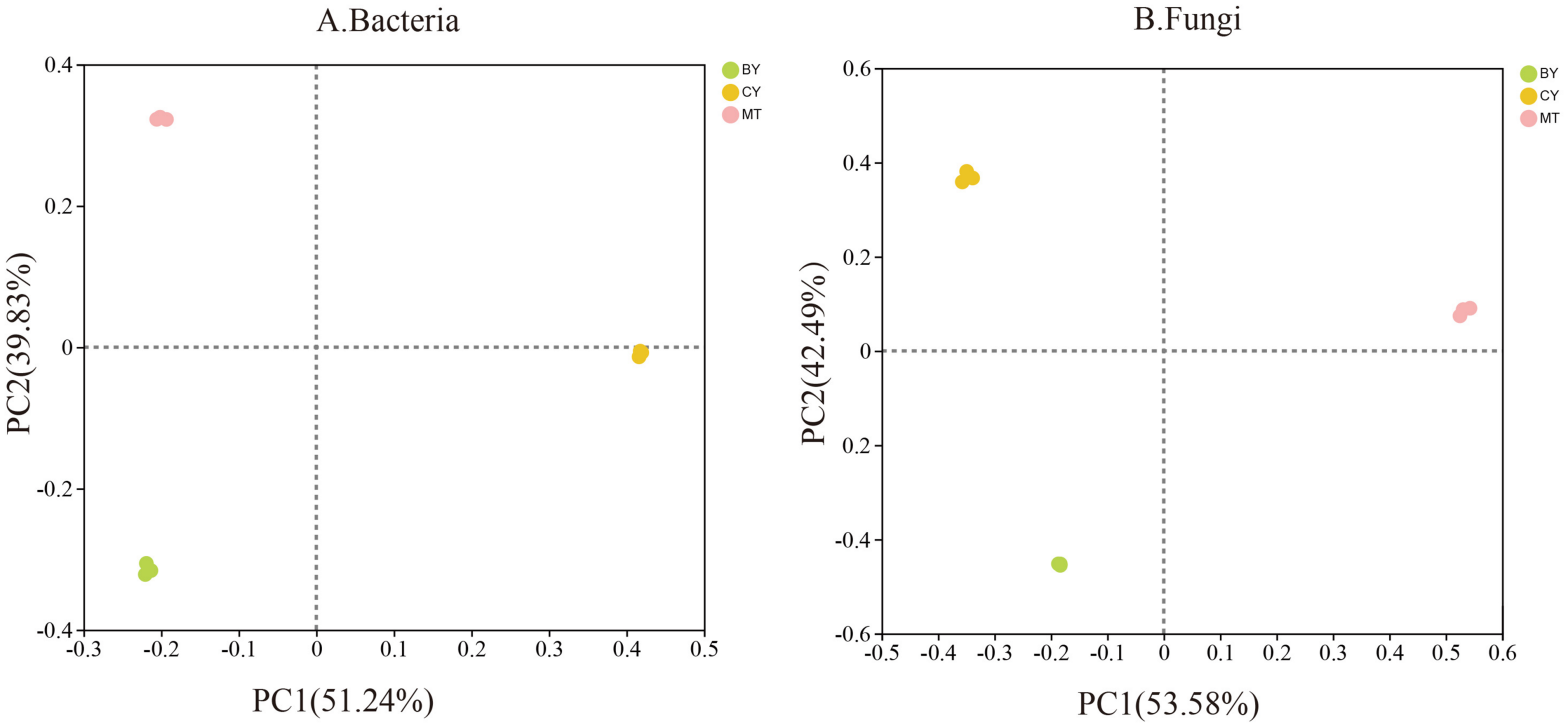
Principal component analysis of Bacteria **(A)**, Fungi **(B)** communities in the rhizosphere soil of *C. tracyanum*.

Based on the principal component analysis results in Figure 3A, PC1 and PC2 accounted for 51.24 and 39.83% of the variation in rhizosphere soil bacterial communities, respectively, with a cumulative contribution of 91.07%. The samples from BY, CY, and MT were distributed in different quadrants and were relatively distant from each other, suggesting significant differences in bacterial community composition among the three sites. Similarly (Figure 3B), PC1 and PC2 explained 53.58 and 42.49% of the variation in rhizosphere soil fungal communities, respectively, with a cumulative contribution of 96.07%. The BY, CY, and MT samples were again located in different quadrants and were widely separated, indicating significant differences in fungal community composition among the three sites. These results highlight considerable differences in the microbial community structure of rhizosphere soil in *C. tracyanum* from different locations.

### 3.3 Structural analysis of rhizosphere soil microbial communities

The genomic DNA sequencing results of rhizosphere soil microorganisms revealed that the rhizosphere soil from the three sample sites contained 1,535 bacterial OTUs, classified into 17 phyla, 50 classes, 93 orders, 162 families, 337 genera, and 462 species. Eight hundred seventy-one fungal OTUs belonging to 15 phyla, 49 classes, 107 orders, 215 families, 392 genera, and 545 species were identified.

**Figure 4:**
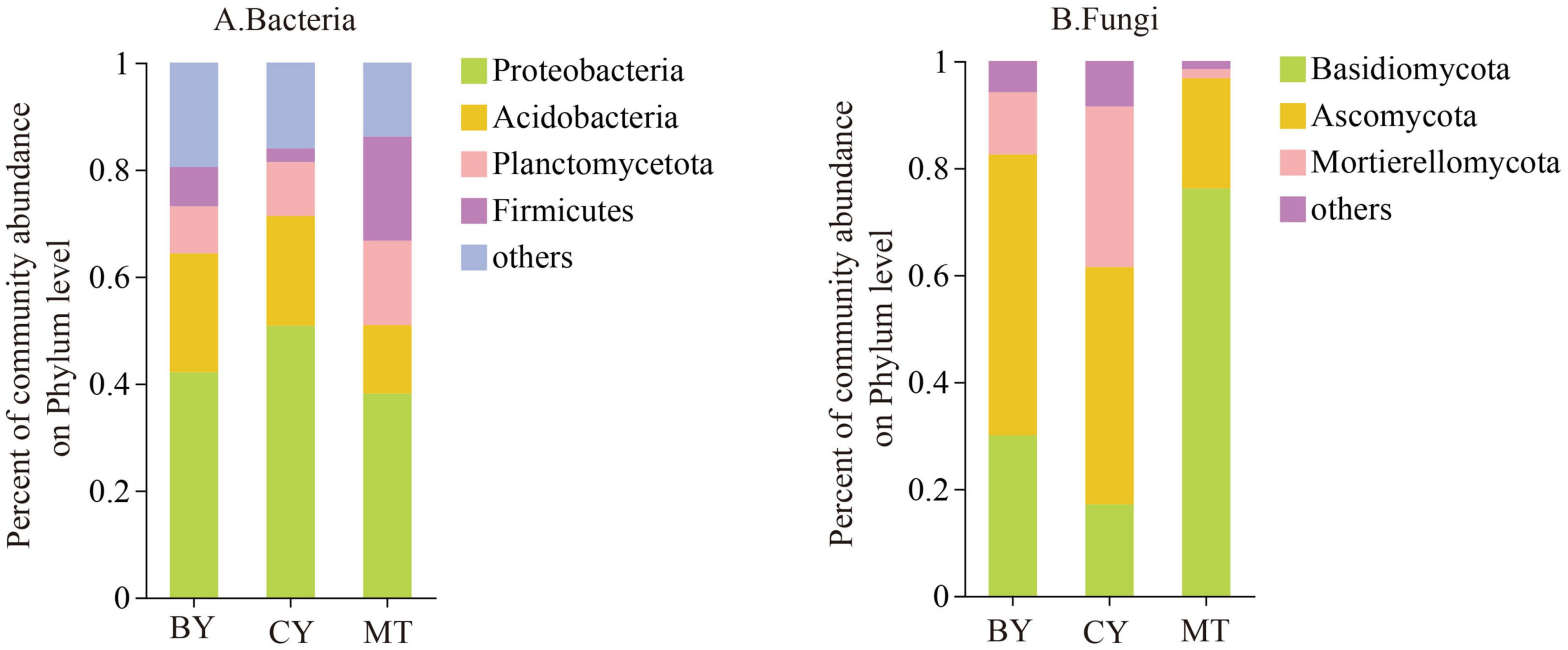
Composition of Bacteria **(A)**, Fungi **(B)** communities in the rhizosphere soil of *C. tracyanum* at the phylum level.

At the phylum level, as shown in Figure 4A, bacterial samples from MT, CY, and BY with a relative abundance greater than 1% were annotated to five phyla, four of which were accurately classified: Proteobacteria, Acidobacteria, Planctomycetota, and Firmicutes. Specifically, bacterial samples from the rhizosphere soil of BY were classified into four phyla: Proteobacteria (41.65%), Acidobacteria (21.55%), Planctomycetota (8.70%), and Firmicutes (7.58%). Bacterial samples from CY were classified into Proteobacteria (51.00%), Acidobacteria (20.25%), Planctomycetota (9.80%), and Firmicutes (3.25%). Similarly, bacterial samples from MT were classified into Proteobacteria (37.32%), Acidobacteria (11.39%), Planctomycetota (14.71%), and Firmicutes (18.83%). These results indicate that Proteobacteria is the dominant bacterial phylum in the rhizosphere soil of *C. tracyanum* across all three sites. However, significant differences exist in their relative abundances and the composition and relative abundances of the secondary dominant phyla (Figure 4B).

The fungal samples from MT, CY, and BY were classified into three phyla: Basidiomycota, Ascomycota, and Mortierellomycota. Among them, the fungal samples from BY were classified into Basidiomycota (30.01%), Ascomycota (52.51%), and Mortierellomycota (11.62%). In CY, the fungal samples were classified into Basidiomycota (17.10%), Ascomycota (44.39%), and Mortierellomycota (30.01%). In MT, the fungal samples were classified into Basidiomycota (76.20%), Ascomycota (20.58%), and Mortierellomycota (1.71%). These findings suggest that Ascomycota is the dominant fungal phylum in the rhizosphere soil of *C. tracyanum* from BY and CY, whereas Basidiomycota is the dominant phylum in MT. Additionally, there are substantial variations in the relative abundances of fungal phyla among the three sites.

**Figure 5:**
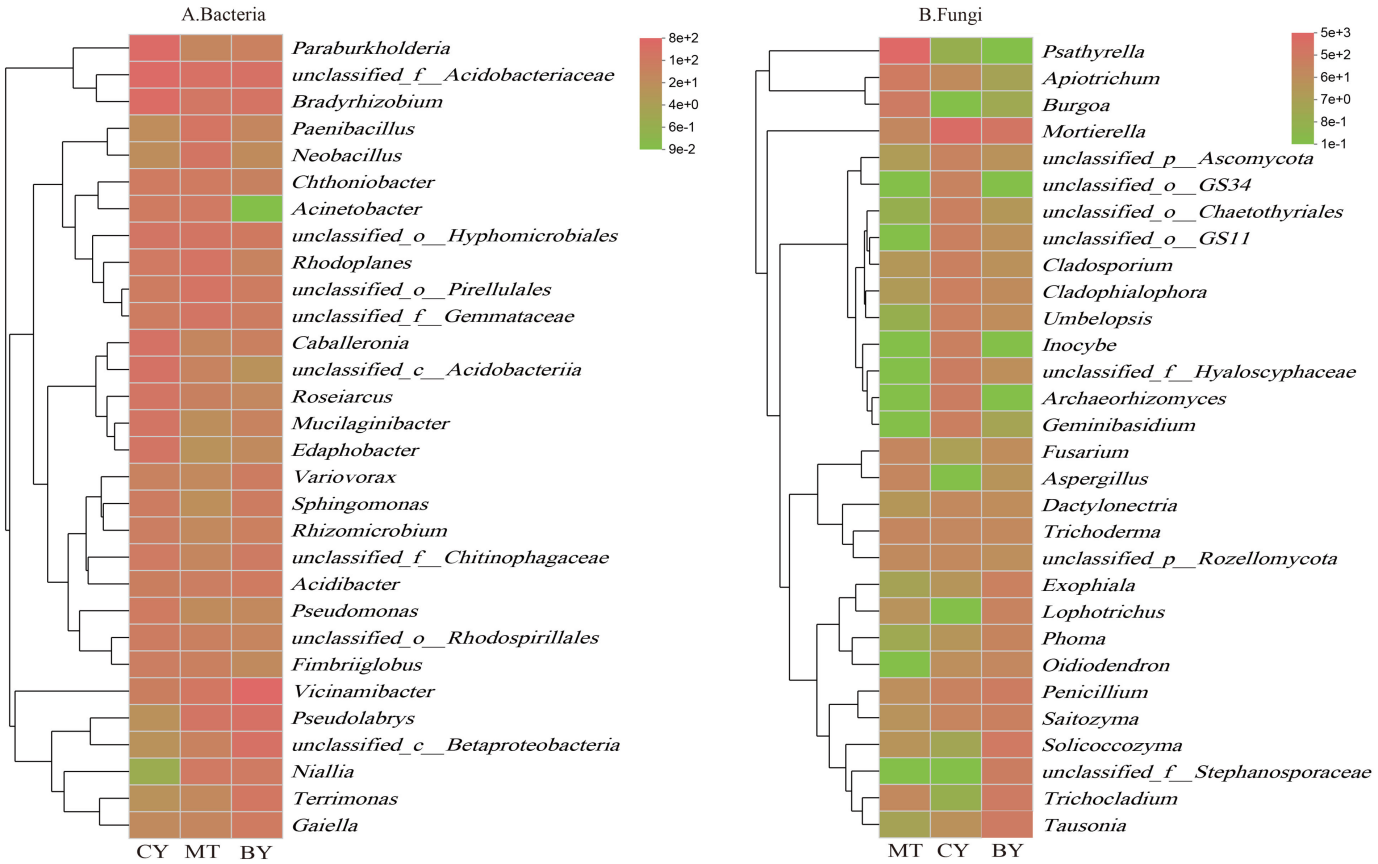
Composition of Bacteria **(A)**, Fungi **(B)** communities in the rhizosphere soil of *C. tracyanum* at the genus level.

At the genus level, as shown in Figure 5A, in the rhizosphere soil of CY, *unclassified_f__Acidobacteriaceae* was the most dominant bacterial genus, with a relative abundance of 8.72%, followed by *Paraburkholderia* (7.85%) and *Bradyrhizobium* (7.05%). In MT, *unclassified_f__Acidobacteriaceae* was also the most dominant bacterial genus (4.62%), followed by *Rhodoplanes* (3.23%), *unclassified_o__Pirellulales* (3.11%), and *Paenibacillus* (3.08%). In BY, *Vicinamibacter* was the most dominant bacterial genus (10.21%), followed by *unclassified_f__Acidobacteriaceae* (4.10%), *unclassified_c__Betaproteobacteria* (3.99%), *Pseudomonas* (3.89%), and (Figure 5B) *Bradyrhizobium* (3.17%).

In the rhizosphere soil of MT, *Psathyrella* was the most dominant fungal genus (59.49%), followed by *Apiotrichum* (6.75%) and *Burgoa* (6.31%). In CY, *Mortierella* was the most dominant fungal genus (30.00%), followed by *Archaeorhizomyces* (4.66%), *unclassified_f__Hyaloscyphaceae* (4.61%), *Geminibasidium* (4.25%), and *unclassified_o__GS11* (3.57%). In BY, *Mortierella* was again the most dominant fungal genus (11.59%), followed by *Solicoccozyma* (8.54%), *Trichocladium* (6.09%), *Tausonia* (6.05%), and *unclassified_f__Stephanosporaceae* (4.65%).

**Figure 6:**
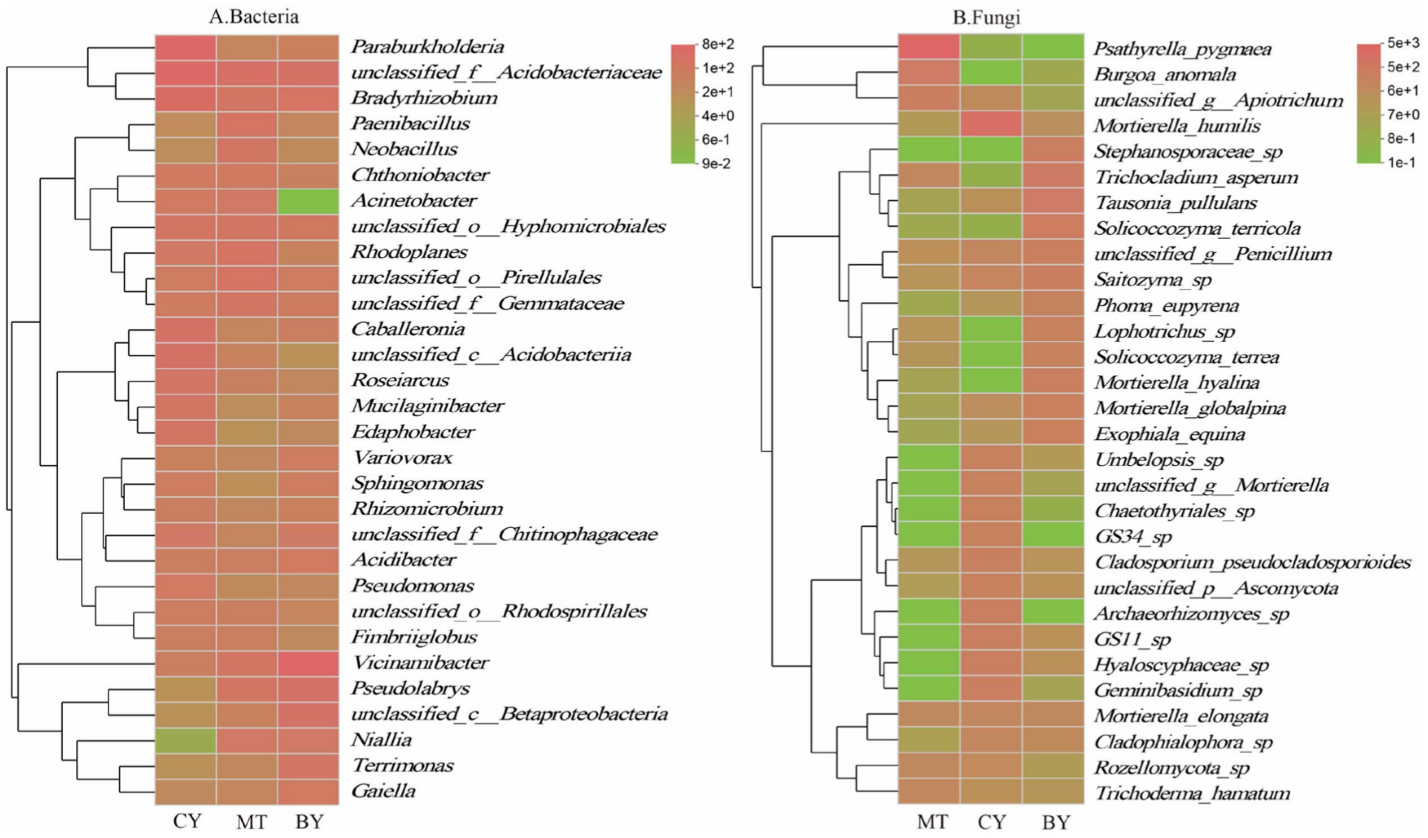
Composition of Bacteria **(A)**, Fungi **(B)** communities in the rhizosphere soil of *C. tracyanum* at the species level.

As shown in Figure 6A, in the rhizosphere soil of CY, *unclassified_f__Acidobacteriaceae* was the most dominant bacterial species (8.72%), followed by *unclassified_g__Bradyrhizobium* (7.05%), *Paraburkholderia fungorum* (5.44%), and *unclassified_c__Acidobacteria* (3.48%). In MT, *unclassified_f__Acidobacteriaceae* was also the most dominant bacterial species (4.62%), followed by *unclassified_o__Pirellulales* (3.14%), *unclassified_g__Rhodoplanes* (3.06%), *Pseudolabrys taiwanensis* (2.88%), and *unclassified_f__Gemmatimonadaceae* (2.85%). In BY, *Vicinamibacter silvestris* was the most dominant bacterial species (10.21%), followed by *unclassified_f__Acidobacteriaceae* (4.10%), *unclassified_c__Betaproteobacteria* (4.00%), *Pseudolabrys taiwanensis* (3.89%), and *unclassified_g__Bradyrhizobium* (3.17%).

As shown in Figure 6B, in the rhizosphere soil of MT, *Psathyrella pygmaea* was the most dominant fungal species (59.50%), followed by *Burgoa anomala* (6.31%) and *unclassified_g__Apiotrichum* (4.28%). In CY, *Mortierella humilis* was the most dominant fungal species (24.29%), followed by *Hyaloscyphaceae* sp. (4.53%), *Geminibasidium* sp. (4.25%), and *GS11* sp. (3.67%). In BY, *Trichoderma hamatum* was the most dominant fungal species (6.09%), followed by *Tausonia pullulans* (6.05%), *Solicoccozyma terricola* (5.87%), and *Stephanosporaceae* sp. (4.65%).

These findings highlight significant differences in the dominant microbial communities and their relative abundances in the rhizosphere soil of *C. tracyanum* across different regions.

### 3.4 Correlation between core microorganisms and soil physicochemical factors

**Figure 7:**
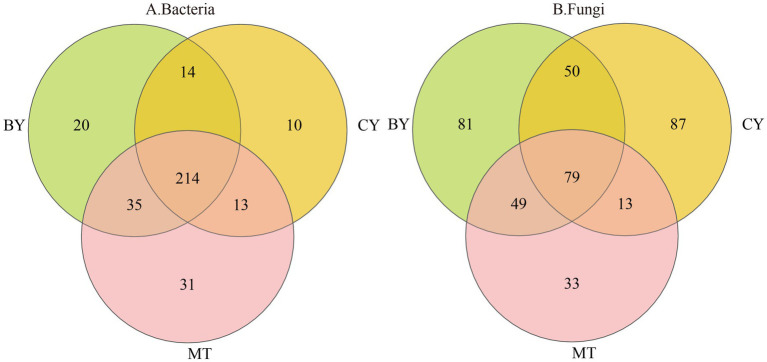
Venn diagrams of Bacteria **(A)**, Fungi **(B)** genus detected in the rhizosphere soil of *C. tracyanum*.

The core microbiome is considered a key component of the fundamental functions of the entire biological community. It is enriched, selected, and inherited through evolutionary processes (Zhang et al., 2020). Core microbial taxa can be analyzed from multiple perspectives, including consortium coexistence, OTU abundance, functional redundancy, and phylogeny. In this study, the coexisting microbial flora in the three plots was defined as the core microbial flora, following the method of Xu Guoqi et al. (Zhao et al., 2014). As shown in Figure 7A, 214 core bacterial species were identified in the rhizosphere soil samples from the three sample sites at the genus level, accounting for 2.86% of the total bacterial community. Among these, the 10 most abundant genera were *unclassified_f__Acidobacteriaceae, Vicinamibacter, Bradyrhizobium, Paraburkholderia, unclassified_o__Hyphomicrobiales, Pseudomonas, unclassified_o__Pirellulales, Rhodoplanes, unclassified_f__Gemmataceae,* and *Caballeronia.*

Similarly, Figure 7B illustrates that 79 core fungal species were identified at the genus level in the rhizosphere soil samples from the three sites, constituting 2.75% of the total fungal community. The 10 most abundant genera were *Psathyrella, Mortierella, Solicoccozyma, Penicillium, Apiotrichum, Trichocladium, Tausonia, Saitozyma, unclassified_f__Hyaloscyphaceae,* and *Cladophialophora.*

**Figure 8:**
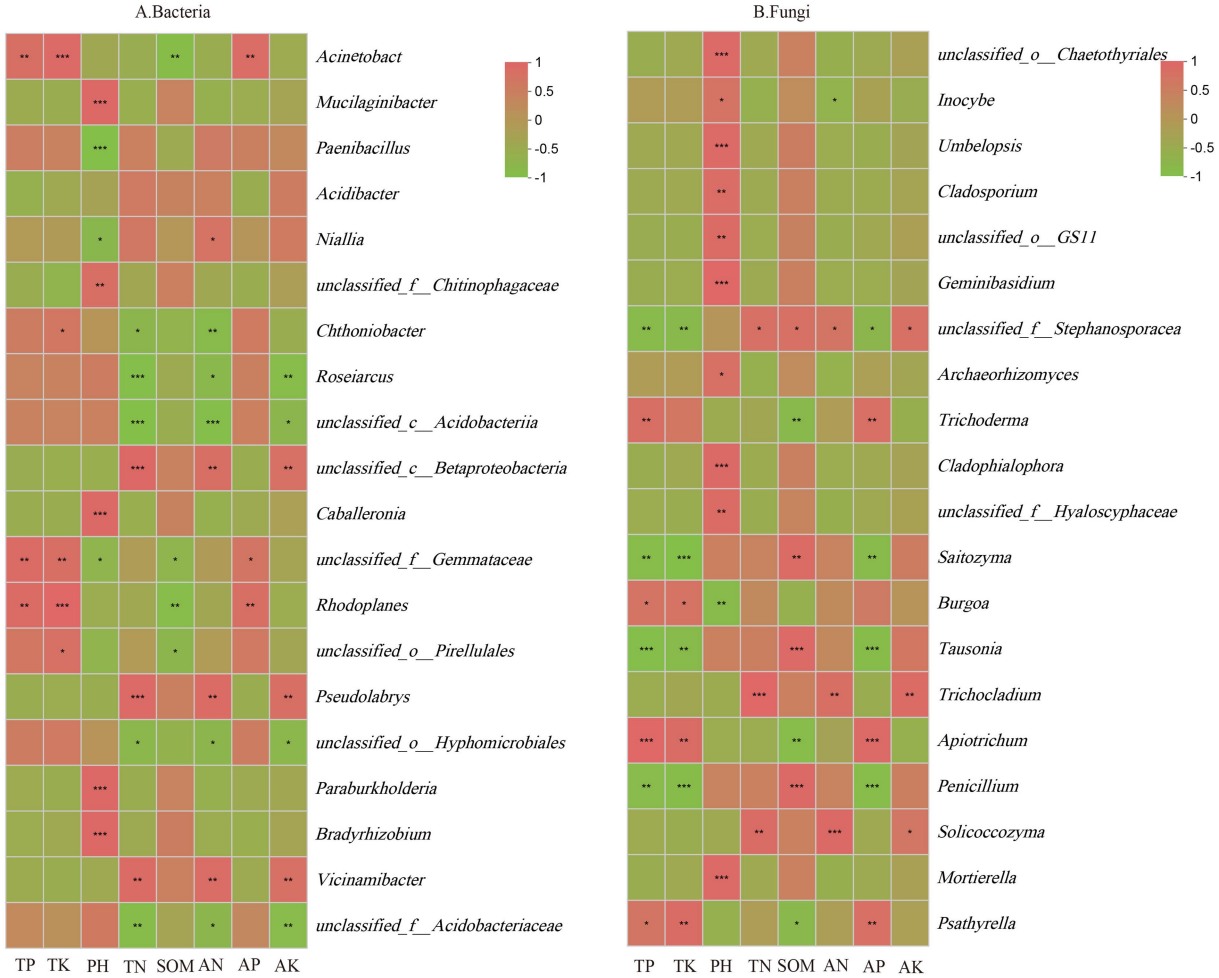
Correlation between soil physicochemical properties and core Bacteria **(A)**, Fungi **(B)** in the rhizosphere soil of *C. tracyanum*. *represents *p* < 0.05, **represents *p* < 0.01, and ***represents *p* < 0.001.

A correlation analysis based on the Pearson algorithm was conducted to examine the relationship between soil physicochemical factors and the 20 most abundant core microbial genera. As shown in Figure 8A, TP content was significantly positively correlated with the relative abundance of *Rhodoplanes, unclassified_f__Gemmataceae,* and *Acinetobacter.* TK content was positively correlated with the relative abundance of five genera, including *unclassified_o__Pirellulales, Rhodoplanes, unclassified_f__Gemmataceae, Chthoniobacter,* and *Acinetobacter.* Soil pH was positively correlated with the relative abundance of *Bradyrhizobium, Paraburkholderia, Caballeronia, unclassified_f__Chitinophagaceae,* and *Mucilaginibacter* but negatively correlated with *unclassified_f__Gemmataceae, Niallia,* and *Paenibacillus.* TN content was positively correlated with *Vicinamibacter, Pseudomonas,* and *unclassified_c__Betaproteobacteria* but negatively correlated with *unclassified_f__Acidobacteriaceae, unclassified_o__Hyphomicrobiales, unclassified_c__Acidobacteria, Roseiarcus,* and *Chthoniobacter.* SOM content was negatively correlated with *unclassified_o__Pirellulales, Rhodoplanes, unclassified_f__Gemmataceae,* and *Acinetobacter.* AN content was positively correlated with *Vicinamibacter, Pseudomonas, unclassified_c__Betaproteobacteria,* and *Niallia* but negatively correlated with *unclassified_f__Acidobacteriaceae, unclassified_o__Hyphomicrobiales, unclassified_c__Acidobacteria, Roseiarcus,* and *Chthoniobacter.* AP content showed a positive correlation with *Rhodoplanes, unclassified_f__Gemmataceae,* and *Acinetobacter.* AK content was positively correlated with *Vicinamibacter, Pseudomonas,* and *unclassified_c__Betaproteobacteria* but negatively correlated with *unclassified_f__Acidobacteriaceae, unclassified_o__Hyphomicrobiales, unclassified_c__Acidobacteria,* and *Roseiarcus.*

As illustrated in Figure 8B, TP content was significantly positively correlated with *Psathyrella, Apiotrichum, Burgoa,* and *Trichoderma* but negatively correlated with *Penicillium, Tausonia, Saitozyma,* and *unclassified_f__Stephanosporaceae.* Similarly, TK content was positively correlated with *Psathyrella, Apiotrichum,* and *Burgoa* but negatively correlated with *Penicillium, Tausonia, Saitozyma,* and *unclassified_f__Stephanosporaceae.* Soil pH was significantly positively correlated with *Mortierella, unclassified_f__Hyaloscyphaceae, Cladophialophora, Archaeorhizomyces, Geminibasidium, unclassified_o__GS11, Cladosporium, Umbelopsis, Inocybe,* and *unclassified_o__Chaetothyriales* but negatively correlated with *Burgoa.* TN content was positively correlated with *Solicoccozyma, Trichocladium,* and *unclassified_f__Stephanosporaceae.* SOM content was positively correlated with *Penicillium, Tausonia, Saitozyma,* and *unclassified_f__Stephanosporaceae* but negatively correlated with *Psathyrella, Apiotrichum,* and *Trichoderma.* AN content was positively correlated with *Solicoccozyma, Trichocladium,* and *unclassified_f__Stephanosporaceae* but negatively correlated with *Inocybe.* AP content was positively correlated with *Psathyrella, Apiotrichum,* and *Trichoderma* but negatively correlated with *Penicillium, Tausonia, Saitozyma,* and *unclassified_f__Stephanosporaceae.* AK content was positively correlated with *Solicoccozyma, Trichocladium,* and *unclassified_f__Stephanosporaceae.* These findings suggest that soil physicochemical properties influence the relative abundance of core microbial communities in rhizosphere soil.

### 3.5 Correlation analysis between rhizosphere soil microbial community structure and soil physicochemical factors

**Figure 9:**
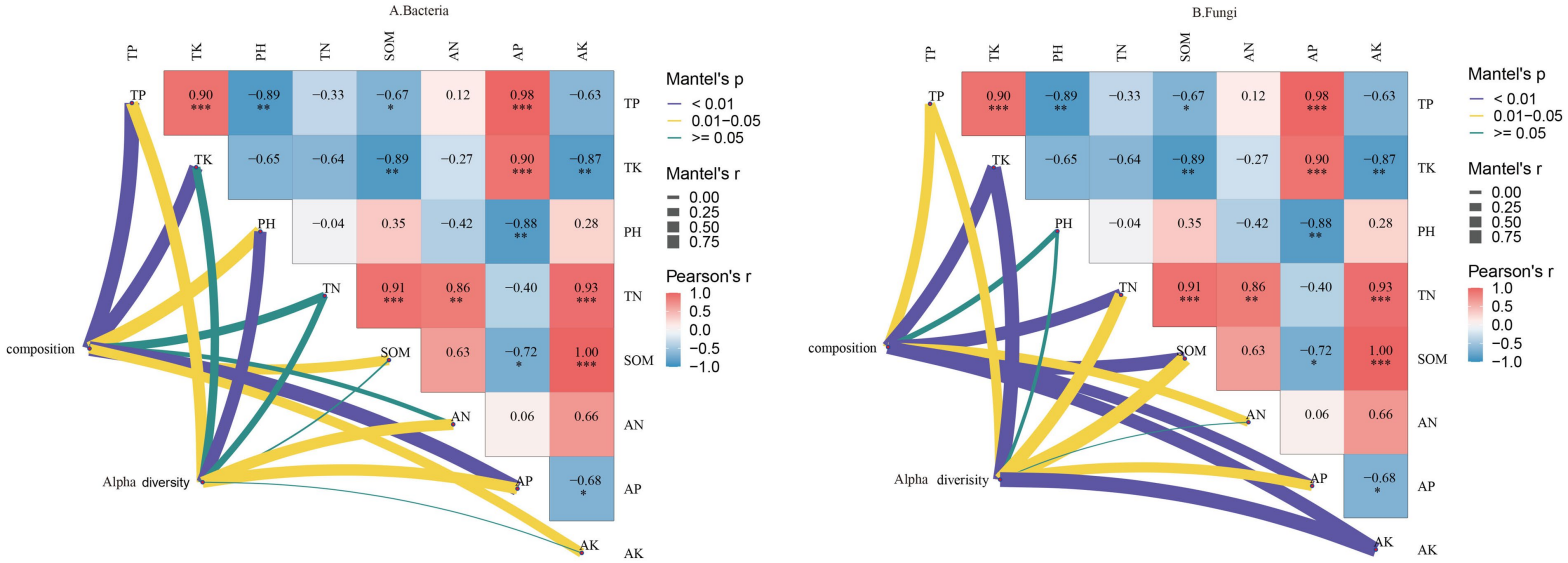
Correlation between the physicochemical properties and composition and α-diversity of Bacteria **(A)**, Fungi **(B)** communities in the rhizosphere soil.

As shown in Figure 9A, the composition of the bacterial community structure in rhizosphere soil was significantly positively correlated with TP, TK, pH, SOM, AP, and AK contents. Additionally, the *α*-diversity of rhizosphere soil bacteria exhibited a significant positive correlation with TP, pH, AN, and AP contents.

As depicted in Figure 9B, the fungal community structure in rhizosphere soil was positively correlated with TP, TK, TN, SOM, AN, AP, and AK contents but negatively correlated with pH. The α-diversity of rhizosphere soil fungi was significantly negatively correlated with pH and AN contents but positively correlated with other environmental factors. These results indicate that TP and AP contents influence microbial diversity in rhizosphere soil.

## 4 Discussion

This study employed high-throughput sequencing technology to systematically analyze the microbial communities across various regions to gain a deeper and more comprehensive understanding of the microbial community composition in the rhizosphere soil of *C. tracyanum*. Furthermore, it examined the relationship between rhizosphere soil and soil physicochemical factors, aiming to uncover potential correlations between rhizosphere microorganisms and these factors.

Soil microorganisms are vital to soil ecosystems, with bacteria and fungi representing the largest biodiversity reservoirs in terrestrial environments. The diversity of these microorganisms is widely recognized as a crucial indicator of soil quality and plant growth and development (Gao et al., 2019; Tan et al., 2021). As the most dynamic component of soil, microorganisms play a pivotal role in material transport, energy flow, and various intricate biochemical reactions. This study revealed that the microbial community structure and diversity in the rhizosphere soil of *C. tracyanum* varied across the three sample sites. The BY, CY, and MT samples contained 565 bacterial OTUs and 86 fungal OTUs, accounting for 7.56 and 3.00% of the total, respectively. The diversity and abundance of soil microorganisms differed among the regions, with the BY sample site exhibiting the highest Chao1 and Shannon indices, suggesting the greatest microbial abundance and diversity in the rhizosphere soil of *C. tracyanum* within the study area. The physicochemical properties of rhizosphere soil influence microbial community composition and diversity. Wang C.Y. et al., through an analysis of microbial community structures in 203 soil samples collected from major grain-producing regions of Northeast China, found that pH is the most significant environmental factor affecting the distribution of bacterial and fungal communities (Wei et al., 2022). Previous studies have indicated that soil nutrient content, pH, and moisture levels are critical in shaping bacterial community structures in the rhizosphere soil of *Salsola passerina* (Zhang et al., 2022). Additionally, soil pH, nitrogen, and phosphorus levels have been shown to influence bacterial community structure and diversity in the rhizosphere soil of *Mirabilis himalaica* (Xi et al., 2022). Li et al. (2020) identified a strong relationship between the physicochemical properties of rhizosphere soil and variations in bacterial community structures in the rhizosphere soil of *Panax ginseng* across different cultivation periods. Similarly, Li and Liu (2019) demonstrated that soil environmental factors and plant species significantly influence the composition and diversity of microbial communities in the rhizosphere soil of medicinal plants. This study also revealed differences in the physicochemical properties and microbial diversity of *C. tracyanum* rhizosphere soil across the three sampled sites. Notably, TP and AP contents were found to be crucial in shaping microbial diversity. Burns et al. (2015) further confirmed that plant species, soil physicochemical properties, and spatial factors significantly impact the diversity of soil microbial communities. In conclusion, soil physicochemical factors and spatial variability are key drivers of microbial community diversity in the rhizosphere soil of different Tibetan regions.

From the perspective of bacterial communities, the dominant bacterial phyla in the rhizosphere soil of *C. tracyanum* were Proteobacteria and Acidobacteria, followed by Planctomycetota and Firmicutes. Ting (n.d.) reported that the predominant bacterial phyla in the rhizosphere soil of rapeseed (*Brassica napus*) included Proteobacteria, Bacteroidetes, Acidobacteria, Actinobacteria, Gemmatimonadetes, Planctomycetes, and Firmicutes. These findings are consistent with our study, which identified Proteobacteria and Firmicutes as dominant taxa. Proteobacteria and Firmicutes play key roles in nitrogen fixation, soil remediation, and organic matter degradation, improving plant quality and stress resistance (Song et al., 2016; Guo et al., 2017). Acidobacteria are involved in the decomposition of organic matter and enhance plant resilience. Enriching these four bacterial phyla in the rhizosphere soil of Tibet suggests their involvement in the growth, development, and stress resistance of *C. tracyanum* in this region. Within the fungal communities, the dominant phyla in the rhizosphere soil of *C. tracyanum* were Basidiomycota, Ascomycota, and Mortierellomycota. A study by Jie et al. (2021) on *Cymbidium ensifolium* found that the predominant fungal phyla in rhizosphere soil were Basidiomycota, Ascomycota, and Mortierellomycota, consistent with our findings. Research indicates that Ascomycota and Basidiomycota are the primary fungal groups involved in decomposing SOM and play essential roles in the nitrogen cycle, mycorrhizal formation, and plant growth and development (Yelle et al., 2010). The enrichment of Ascomycota and Basidiomycota in *C. tracyanum* rhizosphere soil suggests their significant contribution to the growth and development of this species in Tibetan regions.

Studies have shown that core microbial groups are crucial in maintaining soil ecological stability and resisting pathogen invasion (Gómez et al., 2017; Schulz-Bohm et al., 2018; Marfetán et al., 2023). This study defined microorganisms commonly found in *C. tracyanum* across the three sample sites as core microbial communities. Regarding microbial composition, 214 core bacterial communities and 79 core fungal communities were identified in the rhizosphere soil samples. The number of core bacterial communities was significantly higher than that of core fungal communities. The core bacterial communities in the rhizosphere soil of *C. tracyanum* included *unclassified_f__Acidobacteriaceae, Vicinamibacter, Bradyrhizobium, Paraburkholderia, unclassified_o__Hyphomicrobiales, Pseudomonas, unclassified_o__Pirellulales, Rhodoplanes, unclassified_f__Gemmataceae,* and *Caballeronia*. Meanwhile, the core fungal communities consisted of *Psathyrella, Mortierella, Solicoccozyma, Penicillium, Apiotrichum, Trichocladium, Tausonia, Saitozyma, unclassified_f__Hyaloscyphaceae,* and *Cladophialophora*. Certain core microbial groups serve essential biological functions. For instance, *Mortierella* fungi can solubilize phosphorus and exhibit inhibitory effects on pathogenic fungi (Marfetán et al., 2023; Zhang et al., 2011). *Penicillium* promotes plant growth (Baron et al., 2020; Khan et al., 2008), while *Cladosporium* species can produce indole diterpenoid alkaloids (Han et al., 2021). *Bradyrhizobium*, a nitrogen-fixing bacterium in plant-associated soil (Sun et al., 2012), facilitates the transport of mineral nutrients, promotes soil aggregate formation, enhances soil structure, and suppresses soil-borne diseases (Azarias Guimarães et al., 2012). Lin et al. isolated four *Pseudomonas* strains with the phosphorus-solubilizing ability and assessed their effectiveness using the vanadium molybdate blue colorimetric method and pot experiments. The results indicated that these phosphorus-solubilizing bacteria not only significantly increased the height and aboveground dry weight of *Elymus dahuricus* but also had a positive impact on AN and AP levels in the soil (Lin et al., 2022). Additionally, *Rhodoplanes* species possess nitrogen-removal capabilities and can degrade organic compounds and certain refractory nitrogen compounds (Chen et al., 2016). These findings suggest that the rhizosphere soil of *C. tracyanum* harbors a diverse array of core microbial communities with significant biological functions that contribute to soil health and plant growth.

## 5 Conclusion

This study utilized high-throughput sequencing technology to gain initial insights into the bacterial and fungal diversity of the rhizosphere soil of *C. tracyanum*. The findings revealed significant variations in microbial diversity and community composition across different sample sites, with the rhizosphere soil at site BY exhibiting the highest microbial richness and diversity. Proteobacteria, Acidobacteria, Planctomycetota, and Firmicutes were identified as the dominant bacterial phyla, while Ascomycota and Basidiomycota were the predominant fungal phyla in the rhizosphere soil of *C. tracyanum*. Notably, many of these microbial groups contribute to soil fertility and plant growth. Correlation analysis indicated that soil physicochemical properties significantly influence microbial diversity and population abundance in the rhizosphere of *C. tracyanum*. Specifically, high levels of TP and AP in the soil facilitate microbial metabolism and growth. The findings of this study provide a scientific foundation for the effective utilization of rhizosphere microorganisms, offering a theoretical basis for the standardized artificial cultivation of *C. tracyanum* and the production of high-quality plants.

## Data Availability

The original contributions presented in the study are included in the article/supplementary material, further inquiries can be directed to the corresponding author/s.

## Footnotes

^1^ http://www.i-ranger.com
